# An Investigation of Second-Year Medical Students’ Use of Outside Resources at Two Institutions

**DOI:** 10.1007/s40670-024-02243-1

**Published:** 2024-12-06

**Authors:** Andrea Berry, Anna Campbell, Danxun Li, Curt Bay, Uzoma Ikonne

**Affiliations:** 1 Continuous Professional Learning, Office of Faculty Affairs, University of Central Florida College of Medicine, Orlando, FL 32827 USA; 2 Department of Anatomy A.T. Still University, School of Osteopathic Medicine in Arizona, 5850 E. Still Circle, Mesa, AZ 85206 USA; 3 Eastern Virginia Medical School, Norfolk, VA 23507 USA; 4 Department of Interdisciplinary Health Sciences, Arizona School of Health Sciences, A.T. Still University, 5850 E. Still Circle, Mesa, AZ USA; 5 Department of Biomedical and Translational Sciences, Eastern Virginia Medical School, 700 W. Olney Rd, Norfolk, VA 23507 USA; 6 Fine Family Academy of Educators, Eastern Virginia Medical School, Norfolk, VA 23507 USA

**Keywords:** External resources, Non-commercial resources, Commercial resources, Self-regulated learning, Outside resources

## Abstract

**Introduction:**

Medical students have an unprecedented number of study resources available to use. There is a shift in the frequency of student resource use, particularly outside resources not provided by the academic institution, as students progress through the curriculum. This may reflect how individual students develop as self-regulated learners. The purpose of the current study was to evaluate and compare medical student resource use at two institutions.

**Materials and Methods:**

This is a mixed-methods, cross-sectional study that examines factors that are associated with outside resource use and frequency of resource use for second-year medical students across two institutions. A questionnaire was sent to second-year medical students at ATSU-School of Osteopathic Medicine and Eastern Virginia Medical School. Mann–Whitney tests were used to compare Likert-type responses between institutions. A thematic analysis was used to validate and expand on the qualitative dataset.

**Results:**

Students across institutions are using outside resources frequently. We observed similar influence of factors for the use of outside resources such as preparing for licensing exams across institutions. EVMS students were more likely to be influenced by academic support staff and to use outside resources to prepare for course exams than ATSU students. Differences were noted when comparing the use of specific resources such as transcripts, self-generated student resources, and online resources generated by peers. Further, EVMS students more frequently used outside resources to study for disciplines such as physiology, microbiology, and pathology compared to ATSU students.

**Conclusions:**

The observation that students across both institutions are influenced similarly to use outside resources to prepare for licensing exams is expected. However, we did observe some differences which could be explained by variations in curriculum and organizational features or programs at each institution. The results from the current study are consistent with what has been observed in previous studies. Establishing a better understanding of how students use outside resources will enable faculty and institutions to help students develop as self-regulated learners.

**Supplementary Information:**

The online version contains supplementary material available at 10.1007/s40670-024-02243-1.

## Introduction

Medical education has experienced profound shifts in the past decade as curricular content has become ubiquitous. In addition to increased availability of tools that facilitate learning, materials created by medical school faculty and by non-commercial and commercial providers present students with choices for how they obtain, retain, and practice foundational knowledge. Evidence suggests that many medical students are abandoning traditional classroom learning models [1] in favor of recorded lectures for learning course content [2]. Further, students often turn to numerous virtual programs that are available anywhere and anytime, usually for a minimal fee [3]. This shift, which likely explains the proliferation of virtual resources, raises questions about why students turn to externally created content as secondary and sometimes primary study tools.

Current literature suggests a range of reasons for student use of externally created study content [4–6]. Because these tools are primarily oriented to board preparation, student use of externally created question banks has been positively correlated with licensing exam performance [4]. Ease of access and perceived quality of content are also important [5, 6]. Although such research provides important observations about student behaviors [4–6], it does not fully identify the root causes that drive these student choices.

Self-regulated learning theory (SRL) [7–9] may be one way to investigate how and why students select resources for learning. SRL is a prevalent concept in the medical education literature as it represents an important skill that aids in lifelong learning, academic success, and clinical competence [10, 11]. The theory states that students autonomously develop a process, based on their individual learning goals, to acquire knowledge and meet performance expectations. This is reinforced by the fact that students are turning to outside resources to enhance the efficiency of learning foundational concepts [12]. Particularly, interventions that focus on goal setting, monitoring, reflection, and metacognition can influence self-regulated learning levels in students [11]. Through this lens, medical educators can assess important factors in the learning environment and organizational features that may influence student behaviors and motivation.

The purpose of the current study was to determine if student resource use is independent of institutional culture and curriculum. Further, examining why and how often medical students selected resources that were instructor created, student generated, or outside the formal medical school infrastructure. We selected A.T. Still University – School of Osteopathic Medicine in Arizona (ATSU) school and Eastern Virginia Medical School (EVMS) for multiple reasons. First, they are schools that represent two major pathways, allopathic and osteopathic, for students to become a physician in the USA. Also, their class sizes and curricular segments are comparable. However, second-year students at ATSU are part of a distributed educational model where they reside and learn at community partner sites such as community health centers, whereas the students at EVMS are on their home campus.

## Methods

The current study used a mixed-methods, triangulation design within a postpositivist epistemological framework [13], so our study survey included primarily quantitative items and two qualitative items. This method was selected to investigate the students’ objective reality in relation to their use of outside resources while allowing for an interpretive view of the phenomenon [14]. We also included qualitative data in our study to validate and expand on the quantitative results.

### Participants

Second-year students from the A.T. Still University’s School of Osteopathic Medicine in Arizona (ATSU) and Eastern Virginia Medical School (EVMS) were recruited. The local institutional review boards of each school considered the study exempt. Data collection started in October of 2022 with an initial email invitation to participate. Email addresses of students were obtained from each institution’s designated contact in student affairs, academic affairs, or evaluation and assessment offices. The invitation included an explanation of the study and a link to the electronic survey. Students were informed that by clicking on the link to the survey they provided their consent for participation. Two reminder emails were sent and the survey was closed at the end of January 2023. No identifiable private information was collected on the survey; however, the following demographic data were obtained: the institution where the student was enrolled, age, sex, and highest degree attained. Data were stored in a secure Qualtrics account that used Transport Layer Security encryption for all transmitted data. Data were accessed and analyzed by three study investigators.

### Study Institutions

The medical schools included in the current study had different curricular models. These differences in the curriculum meant that the institutions had different preclerkship learning environments, student cultures, and study resources.

At ATSU, students are on campus for the first academic year and then move to community partner sites for the second year, where didactic learning is combined with clinical experiences. Courses are generally systems focused and occur in 10-week blocks during the first and second years. Throughout their preclerkship coursework, ATSU students take approximately two examinations every 10-week period. Exams have integrated content and Comprehensive Osteopathic Medical Licensing Examination of the United States Level 1 style questions that are primarily created by the course instructors. Although courses in the preclerkship curriculum are pass-fail, numeric grades are provided and tracked for class ranking purposes. Students interact with faculty advisors and student achievement success advisors on a regular basis for academic support and advising. In cases of poor exam performance or course failures, advisement meetings are required.

At EVMS, students are on campus for 2 years for the preclerkship curriculum. Students complete nine modules during that time, and each module has a minimum of two examinations. The examinations include retired National Board of Medical Examiners (NBME) items, and the modules use a pass-fail grading mechanism.

### Survey Design

A survey developed and used in a previous study [15] was adapted for our study. To follow best practices related to survey design, Likert-type responses assessing agreement were revised to assess likelihood. We expanded items that addressed the purposes for using outside resources [16]. Our revised 12-item survey took approximately 15 min to complete and included four demographic questions: one question to identify the likelihood of using outside resources based on the recommended source; one question to identify the likelihood of using outside resources for various learning, studying, and assessment activities; one question to identify the frequency of use of outside resources; one question to identify the frequency of use of outside resources based on basic science disciplines; and two open-ended qualitative questions to assess other reasons for using outside resources and to gather additional comments about the use of outside resources. Participants were not required to respond to every question. Before distribution, the survey was reviewed by faculty and students to ensure face and content validity [16]. The online survey (Appendix) was hosted on the Qualtrics (Seattle, WA) platform.

### Statistical and Thematic Analysis

 Survey responses for students from ATSU and EVMS were summarized using frequency and percentage. Because students did not have to answer every survey question, our analyses included both complete and partially completed surveys. Likert-type responses of likely and extremely likely, almost always true and usually true, and daily and several times a week were grouped as likely for analysis. Means and standard deviations were calculated, and Mann–Whitney tests were used to compare Likert-type responses between students at ATSU and EVMS. A *p* value of 0.05 (two-tailed) was used as the criterion for statistical significance. SPSS version 28 (IBM Corp, Armonk NY) was used for all analyses.

Qualitative data from the two open-ended survey questions were downloaded into Excel (Microsoft Corp, Seattle, WA) and manually reviewed. Using the six steps outlined by Braun and Clarke [17], a single investigator (AB) identified patterns from student responses. Initial codes were generated and then examined to determine connections between codes and themes of broader significance [18]. After initial themes were generated by the investigator, the full research team met to reach consensus regarding the final themes for the qualitative data. We also verified that the naming conventions of our defined themes reflected the data and were related to our research question.

## Results

### Participants

Thirty-six (24.3%) of 148 s-year students from ATSU and 29 (19.3%) of 150 s-year students from EVMS responded to the survey. Of those, 9 men, 21 women, and 1 nonbinary person were from ATSU, and 11 men and 18 women were from EVMS (Table 1). Age and education characteristics were similar for both groups of students.

**Table 1:** Demographic characteristics of second-year medical students who completed the study survey

Demographic characteristic	No. (%)		
	ATSU (*n* = 36)	EVMS (*n* = 29)	Total (*N* = 65)
Age			
20–25 years	12 (38.7)	16 (55.2)	28 (46.7)
26–30 years	14 (45.2)	10 (34.5)	24 (40.0)
Over 30 years	5 (16.1)	3 (10.3)	8 (13.3)
Sex			
Man	9 (29.0)	11 (37.9)	20 (33.3)
Woman	21 (67.7)	18 (62.1)	39 (65.0)
Nonbinary	1 (3.2)	0 (0)	1 (1.7)
Highest degree attained			
Bachelor of arts	4 (12.9)	4 (13.8)	8 (13.3)
Bachelor of science	21 (67.7)	17 (58.6)	38 (63.3)
Master of science	5 (16.1)	7 (24.1)	12 (20.0)
Doctoral degree	0 (0)	1 (3.4)	1 (1.7)
Other	1 (3.2)	0 (0)	1 (1.7)

### Use of Outside Resources Recommended by Others

**Fig. 1:**
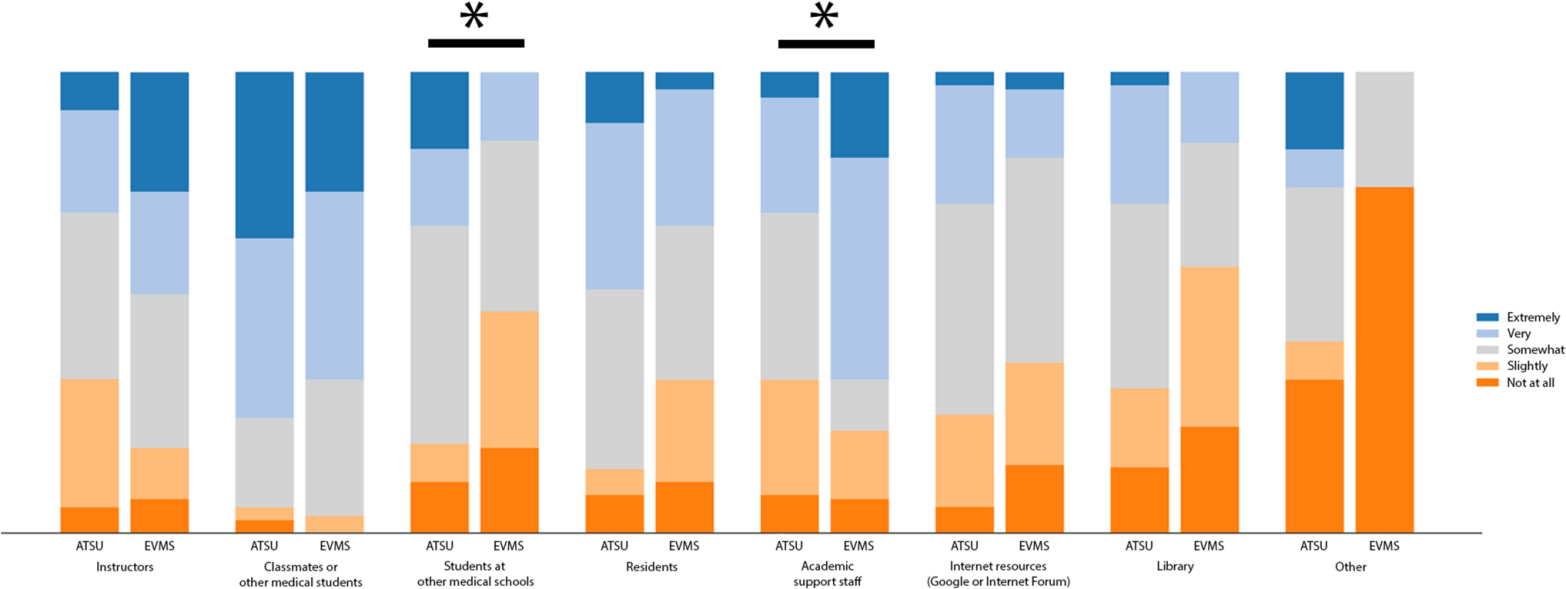
Student reported use outside resources by recommendation. All values are percentage endorsements within each category. *p* < 0.05

Outside resources recommended by classmates or other medical students (27/36, 75.0% ATSU students; 18/27, 66.6% EVMS students) were used most often by both groups (Table 2). However, EVMS students were equally as likely to use resources recommended by academic support staff (18/27, 66.6%). Differences were found between the two groups for outside resources recommended by students at other medical schools (*p* = 0.01) and academic support staff (*p* = 0.02) (Fig. 1).

**Table 2:** Survey responses of second-year medical students regarding use of outside resources recommended by others

Outside resource recommendation	No. (%)					*p* value
	Not at all	Slightly	Somewhat	Very	Extremely	
Instructors						
ATSU (*n* = 36)	2 (5.6)	10 (27.8)	13 (36.1)	8 (22.2)	3 (8.3)	0.06
EVMS (*n* = 27)	2 (7.4)	3 (11.1)	9 (33.3)	6 (22.2)	7 (25.9)	
Classmates or other medical students						
ATSU (*n* = 36)	1 (2.8)	1 (2.8)	7 (19.4)	14 (38.9)	13 (36.1)	0.39
EVMS (*n* = 27)	0 (0)	1 (3.7)	8 (29.6)	11 (40.7)	7 (25.9)	
Students at other medical schools						
ATSU (*n* = 36)	4 (11.1)	3 (8.3)	17 (47.2)	6 (16.7)	6 (16.7)	0.01
EVMS (*n* = 27)	5 (18.5)	8 (29.6)	10 (37.0)	4 (14.8)	0 (0)	
Residents						
ATSU (*n* = 36)	3 (8.3)	2 (5.6)	14 (38.9)	13 (36.1)	4 (11.1)	0.14
EVMS (*n* = 27)	3 (11.1)	6 (22.2)	9 (33.3)	8 (29.6)	1 (3.7)	
Academic support staff						
ATSU (*n* = 36)	3 (8.3)	9 (25.0)	13 (36.1)	9 (25.0)	2 (5.6)	0.02
EVMS (*n* = 27)	2 (7.4)	4 (14.8)	3 (11.1)	13 (48.1)	5 (18.5)	
Internet resources (e.g., Google or internet forum)						
ATSU (*n* = 35)	2 (5.7)	7 (20.0)	16 (45.7)	9 (25.7)	1 (2.9)	0.18
EVMS (*n* = 27)	4 (14.8)	6 (22.2)	12 (44.4)	4 (14.8)	1 (3.7)	
Library						
ATSU (*n* = 35)	5 (14.3)	6 (17.1)	14 (40.0)	9 (25.7)	1 (2.9)	0.07
EVMS (*n* = 26)	6 (23.1)	9 (34.6)	7 (26.9)	4 (15.4)	0 (0)	
Other						
ATSU (*n* = 12)	4 (33.3)	1 (8.3)	4 (33.3)	1 (8.3)	2 (16.7)	0.21
EVMS (*n* = 4)	3 (75.0)	0 (0)	1 (25.0)	0 (0)	0 (0)

### Purpose for Using Outside Resources

**Fig. 2:**
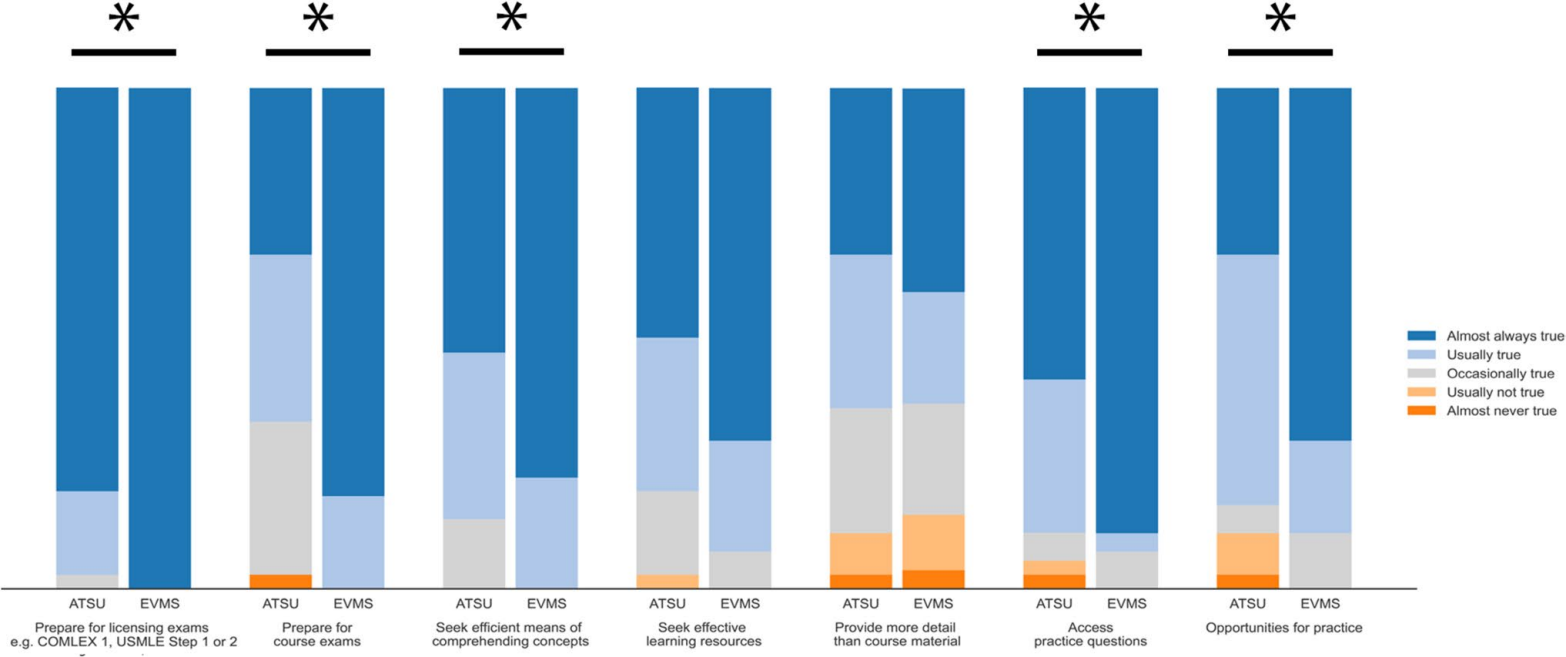
Student reported purpose for using outside resources. All values are percentage endorsements within each category. *p* < 0.05

For most students from both schools, the purpose for using outside resources was to prepare for licensing exams (35/36, 97.3% ATSU students; 27/27, 100% EVMS students), prepare for course exams (24/36, 66.6% ATSU students; 27/27, 100% EVMS students), seek efficient means of comprehending concepts (31/36, 86.1% ATSU students; 27/27, 100% EVMS students), and seek effective learning resources (29/36, 80.6% ATSU students; 25/27, 92.6% EVMS students) (Table 3). Differences were found between the two groups for using outside resources to prepare for licensing exams, prepare for course exams, seek efficient means of comprehending concepts, access practice questions, and have opportunities for practice (all *p* < 0.02) (Fig. 2).

**Table 3:** Survey responses of second-year medical students regarding the purpose for using outside resources

Purpose for using outside resources	No. (%)					*p* value
	Almost always true	Usually true	Occasionally true	Usually not true	Almost never true	
Prepare for licensing exams (e.g., COMLEX 1, USMLE Step 1 or 2)						
ATSU (*n* = 36)	29 (80.6)	6 (16.7)	1 (2.8)	0 (0)	0 (0)	0.01
EVMS (*n* = 27)	27 (100)	0 (0)	0 (0)	0 (0)	0 (0)	
Prepare for course exams						
ATSU (*n* = 36)	12 (33.3)	12 (33.3)	11 (30.6)	0 (0)	1 (2.8)	< 0.001
EVMS (*n* = 27)	22 (81.5)	5 (18.5)	0 (0)	0 (0)	0 (0)	
Seek efficient means of comprehending concepts						
ATSU (*n* = 36)	19 (52.8)	12 (33.3)	5 (13.9)	0 (0)	0 (0)	0.02
EVMS (*n* = 27)	21 (77.8)	6 (22.2)	0 (0)	0 (0)	0 (0)	
Seek effective learning resources						
ATSU (*n* = 36)	18 (50.0)	11 (30.6)	6 (16.7)	1 (2.8)	0 (0)	0.07
EVMS (*n* = 27)	19 (70.4)	6 (22.2)	2 (7.4)	0 (0)	0 (0)	
Provide more detail than course material						
ATSU (*n* = 36)	12 (33.3)	11 (30.6)	9 (25.0)	3 (8.3)	1 (2.8)	0.84
EVMS (*n* = 27)	11 (40.7)	6 (22.2)	6 (22.2)	3 (11.1)	1 (3.7)	
Access practice questions						
ATSU (*n* = 36)	21 (58.3)	11 (30.6)	2 (5.6)	1 (2.8)	1 (2.8)	0.02
EVMS (*n* = 27)	24 (88.9)	1 (3.7)	2 (7.4)	0 (0)	0 (0)	
Have a framework for understanding content						
ATSU (*n* = 36)	14 (38.9)	13 (36.1)	7 (19.4)	2 (5.6)	0 (0)	0.13
EVMS (*n* = 27)	14 (51.9)	10 (37.0)	3 (11.1)	0 (0)	0 (0)	
Have an interactive experience						
ATSU (*n* = 36)	8 (22.2)	12 (33.3)	9 (25.0)	6 (16.7)	1 (2.8)	0.76
EVMS (*n* = 27)	8 (29.6)	5 (18.5)	6 (22.2)	6 (22.2)	2 (7.4)	
Receive feedback						
ATSU (*n* = 36)	5 (13.9)	13 (36.1)	12 (33.3)	3 (8.3)	3 (8.3)	0.53
EVMS (*n* = 27)	6 (22.2)	7 (25.9)	4 (14.8)	4 (14.8)	6 (22.2)	
Have opportunities for practice						
ATSU (*n* = 36)	12 (33.3)	18 (50.0)	2 (5.6)	3 (8.3)	1 (2.8)	0.02
VMS (*n* = 27)	19 (70.4)	5 (18.5)	3 (11.1)	0 (0)	0 (0)	

*ATSU* A.T. Still University’s School of Osteopathic Medicine in Arizona, *COMLEX* Comprehensive Osteopathic Medical Licensing Examination of the United States, *EVMS* Eastern Virginia Medical School, *USMLE* United States Medical Licensing Examination

### Frequency of Use of Resources

**Fig. 3:**
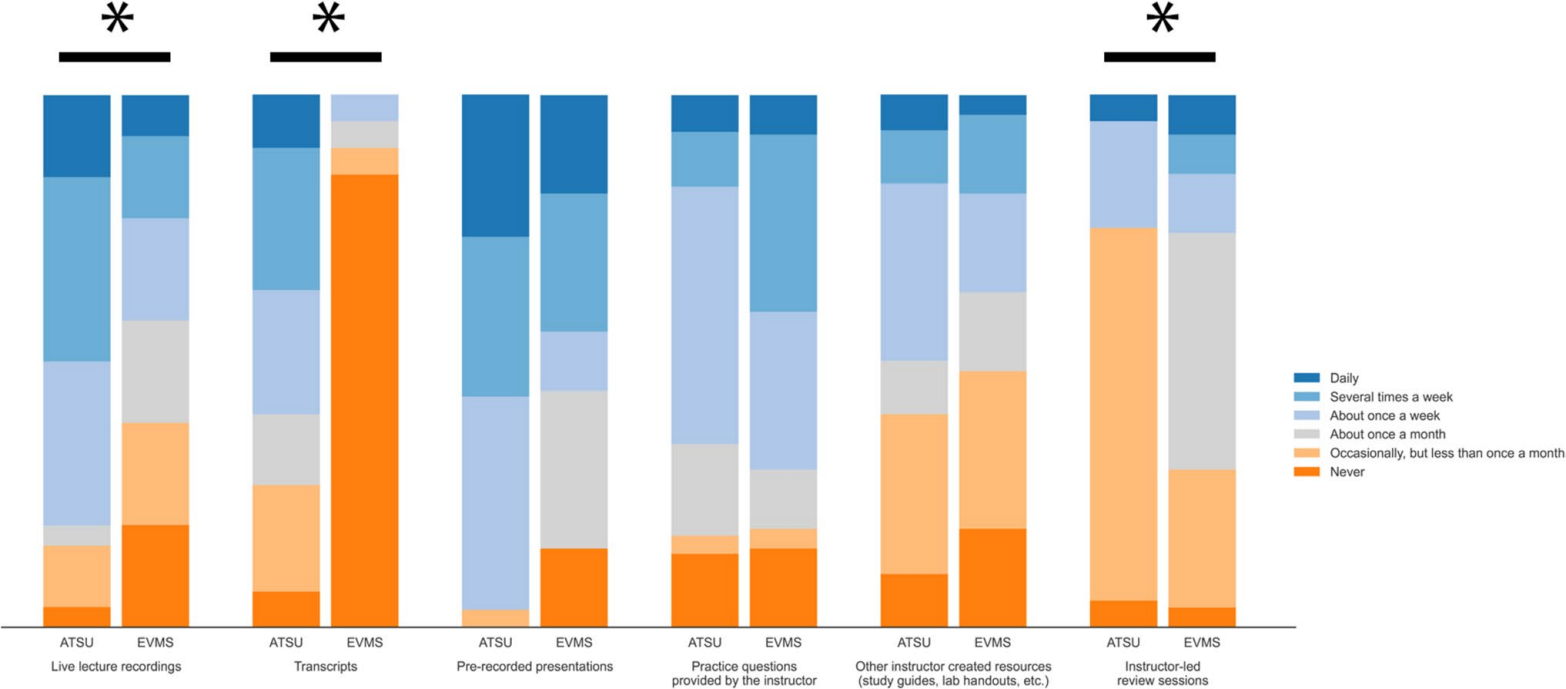
Students reported frequency use of instructor-provided resources. All values are percentage endorsements within each category. *p* < 0.05

For frequency of use of instructor-produced resources, students from both schools most often used instructor slides (21/31, 67.7% ATSU students; 15/27, 55.5% EVMS students) (Table 4). Differences were found between the two groups for using instructor-produced resources of live lecture recordings, transcripts, and instructor-led review sessions (all *p* < 0.02) (Fig. 3).

**Table 4:** Survey responses of second-year medical students for frequency of use of instructor-produced resources

Frequency of use of instructor-produced resources	No. (%)						*p* value
	Never	Occasionally but less than once a month	About once a month	About once a week	Several times a week	Daily	
Instructor slides							
ATSU (*n* = 31)	0 (0)	1 (3.2)	1 (3.2)	8 (25.8)	9 (29.0)	12 (38.7)	0.11
EVMS (*n* = 27)	3 (11.1)	1 (3.7)	3 (11.1)	5 (18.5)	8 (29.6)	7 (25.9)	
Live lecture recordings							
ATSU (*n* = 26)	1 (3.8)	3 (11.5)	1 (3.8)	8 (30.8)	9 (34.6)	4 (15.4)	0.007
EVMS (*n* = 26)	5 (19.2)	5 (19.2)	5 (19.2)	5 (19.2)	4 (15.4)	2 (7.7)	
Transcripts							
ATSU (*n* = 30)	2 (6.7)	6 (20.0)	4 (13.3)	7 (23.3)	8 (26.7)	3 (10.0)	< 0.001
EVMS (*n* = 20)	17 (85.0)	1 (5.0)	1 (5.0)	1 (5.0)	0 (0)	0 (0)	
Prerecorded presentations							
ATSU (*n* = 30)	0 (0)	1 (3.3)	0 (0)	12 (40.0)	9 (30.0)	8 (26.7)	0.05
EVMS (*n* = 27)	4 (14.8)	0 (0.0)	8 (29.6)	3 (11.1)	7 (25.9)	5 (18.5)	
Practice questions provided by the instructor							
ATSU (*n* = 29)	4 (13.8)	1 (3.4)	5 (17.2)	14 (48.3)	3 (10.3)	2 (6.9)	0.26
EVMS (*n* = 27)	4 (14.8)	1 (3.7)	3 (11.1)	8 (29.6)	9 (33.3)	2 (7.4)	
Assigned or recommended reading (e.g., textbook or articles)							
ATSU (*n* = 31)	12 (38.7)	10 (32.3)	5 (16.1)	1 (3.2)	1 (3.2)	2 (6.5)	0.23
EVMS (*n* = 27)	15 (55.6)	8 (29.6)	2 (7.4)	0 (0)	2 (7.4)	0 (0)	
Other instructor-created resources (e.g., study guides, lab handouts)							
ATSU (*n* = 30)	3 (10.0)	9 (30.0)	3 (10.0)	10 (33.3)	3 (10.0)	2 (6.7)	0.58
EVMS (*n* = 27)	5 (18.5)	8 (29.6)	4 (14.8)	5 (18.5)	4 (14.8)	1 (3.7)	
Office hours or other instructor interactions							
ATSU (*n* = 29)	11 (37.9)	12 (41.4)	1 (3.4)	4 (13.8)	1 (3.4)	0 (0)	0.53
EVMS (*n* = 27)	14 (51.9)	8 (29.6)	2 (7.4)	2 (7.4)	0 (0)	1 (3.7)	
Instructor-led review sessions							
ATSU (*n* = 20)	1 (5.0)	14 (70.0)	0 (0)	4 (20.0)	0 (0)	1 (5.0)	0.02
EVMS (*n* = 27)	1 (3.7)	7 (25.9)	12 (44.4)	3 (11.1)	2 (7.4)	2 (7.4)	

*ATSU* A.T. Still University’s School of Osteopathic Medicine in Arizona, *EVMS* Eastern Virginia Medical School

**Fig. 4:**
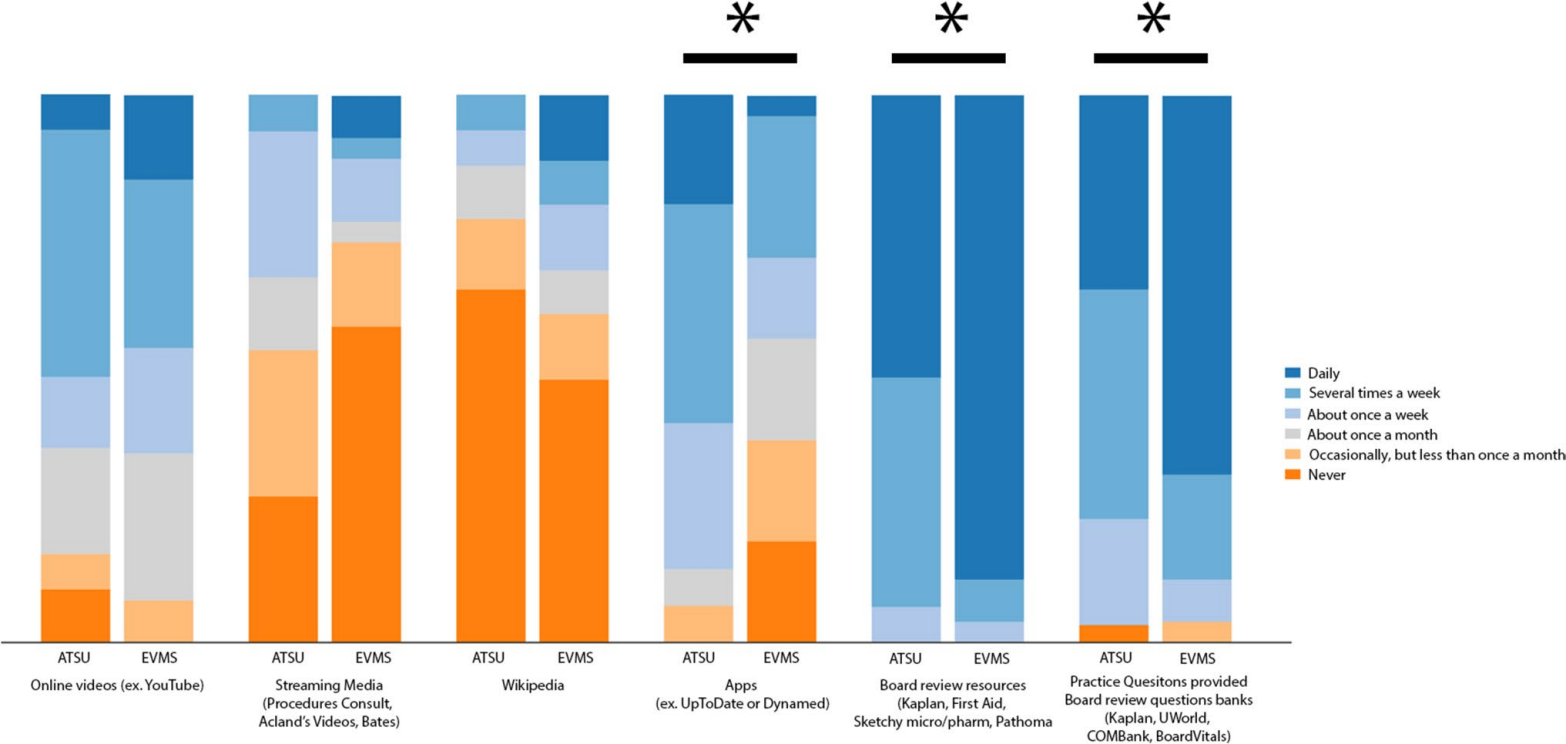
Student reported frequency of use board review resources and applications. All values are percentage endorsements within each category. *p* < 0.05

For frequency of use of outside resources, students from both schools most often used board review resources (29/31, 93.5% ATSU students; 25/26, 96.2% EVMS students) and practice questions provided in board review question banks (24/31, 77.4% ATSU students; 23/26, 88.4% EVMS students) (Table 5). Students from EVMS used these two resources more frequently than students from ATSU (both *p* = 0.01). Students from EVMS used Apps (8/27, 29.6%) less frequently than ATSU students (18/30, 60.0%, *p* = 0.01) (Fig. 4).

**Table 5:** Survey responses of second-year medical students for frequency of use of outside resources

Frequency of use of outside resources	No. (%)						*p* value
	Never	Occasionally but less than once a month	About once a month	About once a week	Several times a week	Daily	
Supplemental textbooks or articles (i.e., not assigned by the instructor)							
ATSU (*n* = 29)	5 (17.2)	12 (41.4)	5 (17.2)	4 (13.8)	2 (6.9)	1 (3.4)	0.01
EVMS (*n* = 26)	15 (57.7)	6 (23.1)	1 (3.8)	2 (7.7)	2 (7.7)	0 (0)	
General search engines (e.g., Google)							
ATSU (n = 31)	0 (0)	6 (19.4)	1 (3.2)	5 (16.1)	7 (22.6)	12 (38.7)	0.88
EVMS (*n* = 26)	2 (7.7)	2 (7.7)	1 (3.8)	5 (19.2)	6 (23.1)	10 (38.5)	
Literature database (e.g., PubMed)							
ATSU (*n* = 31)	4 (12.9)	6 (19.4)	5 (16.1)	10 (32.3)	4 (12.9)	2 (6.5)	0.35
EVMS (*n* = 27)	6 (22.2)	8 (29.6)	2 (7.4)	6 (22.2)	3 (11.1)	2 (7.4)	
Library website search							
ATSU (*n* = 31)	7 (22.6)	7 (22.6)	0 (0)	14 (45.2)	3 (9.7)	0 (0)	0.03
EVMS (*n* = 25)	11 (44.0)	5 (20.0)	5 (20.0)	3 (12.0)	1 (4.0)	0 (0)	
Online videos (e.g., YouTube)							
ATSU (*n* = 31)	3 (9.7)	2 (6.5)	6 (19.4)	4 (12.9)	14 (45.2)	2 (6.5)	0.75
EVMS (*n* = 26)	0 (0)	2 (7.7)	7 (26.9)	5 (19.2)	8 (30.8)	4 (15.4)	
Streaming media (e.g., Procedures Consult, Acland’s Video Atlas, Bates)							
ATSU (*n* = 30)	8 (26.7)	8 (26.7)	4 (13.3)	8 (26.7)	2 (6.7)	0 (0)	0.08
EVMS (*n* = 26)	15 (57.7)	4 (15.4)	1 (3.8)	3 (11.5)	1 (3.8)	2 (7.7)	
Wikipedia							
ATSU (*n* = 31)	20 (64.5)	4 (12.9)	3 (9.7)	2 (6.5)	2 (6.5)	0 (0)	0.11
EVMS (*n* = 25)	12 (48.0)	3 (12.0)	2 (8.0)	3 (12.0)	2 (8.0)	3 (12.0)	
Apps (e.g., UpToDate, DynaMed)							
ATSU (*n* = 30)	0 (0)	2 (6.7)	2 (6.7)	8 (26.7)	12 (40.0)	6 (20.0)	0.01
EVMS (*n* = 27)	5 (18.5)	5 (18.5)	5 (18.5)	4 (14.8)	7 (25.9)	1 (3.7)	
Board review resources (e.g., Kaplan, First Aid, Sketchy Micro or Pharm, Pathoma)							
ATSU (*n* = 31)	0 (0)	0 (0)	0 (0)	2 (6.5)	13 (41.9)	16 (51.6)	0.01
EVMS (*n* = 26)	0 (0)	0 (0)	0 (0)	1 (3.8)	2 (7.7)	23 (88.5)	
Practice questions provided in board review question banks (e.g., Kaplan, Uworld, COMBANK, BoardVitals)							
ATSU (*n* = 31)	1 (3.2)	0 (0)	0 (0)	6 (19.4)	13 (41.9)	11 (35.5)	0.01
EVMS (*n* = 26)	0 (0)	1 (3.8)	0 (0)	2 (7.7)	5 (19.2)	18 (69.2)	
3D models (i.e., printed 3D model, virtual 3D model)							
ATSU (*n* = 30)	19 (63.3)	5 (16.7)	0 (0)	3 (10.0)	3 (10.0)	0 (0)	0.71
EVMS (*n* = 26)	17 (65.4)	5 (19.2)	0 (0)	4 (15.4)	0 (0)	0 (0)	

*ATSU* A.T. Still University’s School of Osteopathic Medicine in Arizona, *EVMS*, Eastern Virginia Medical School

**Fig. 5:**
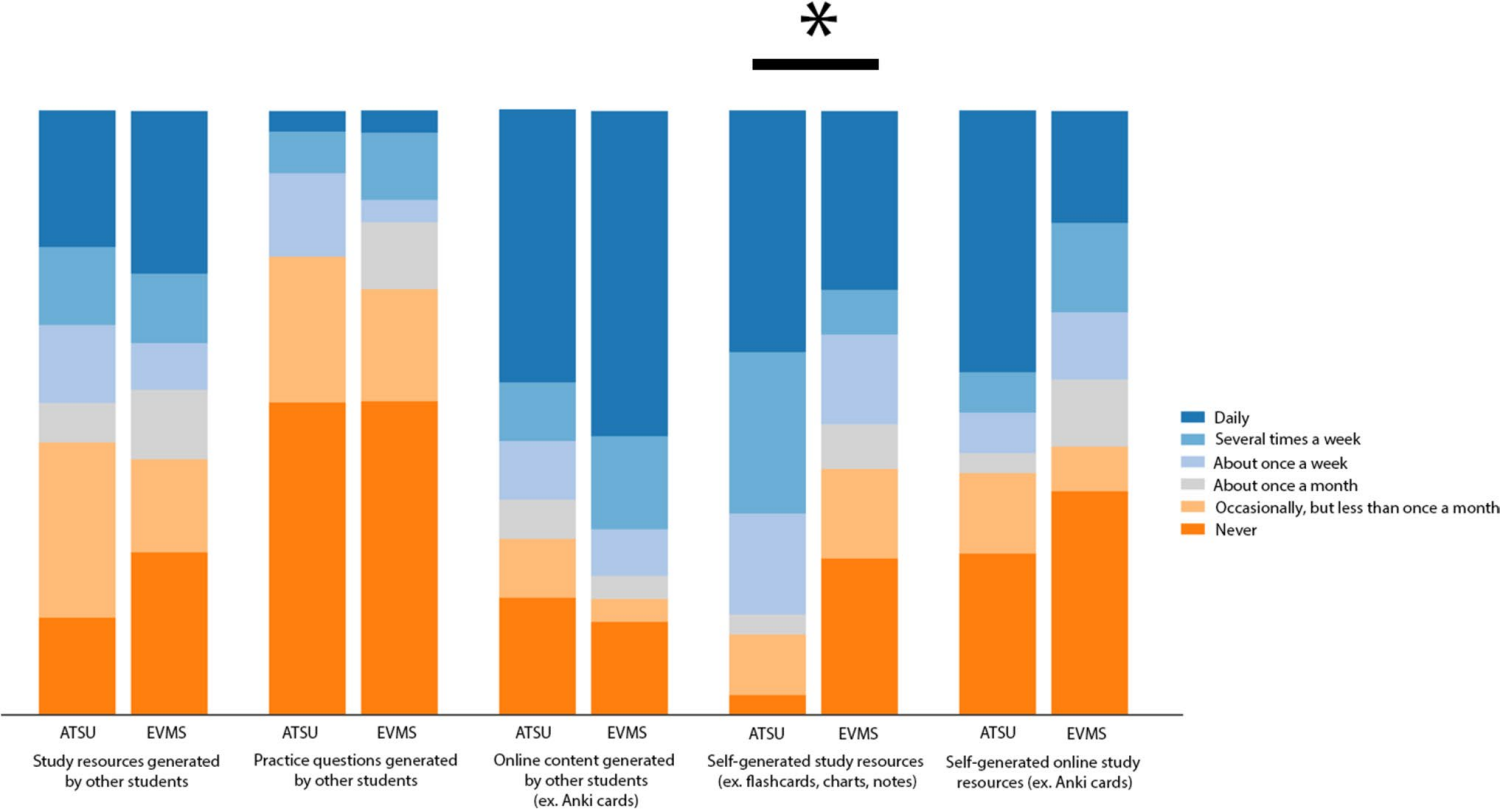
Student reported frequency of use of self and peer-generated resources. All values are percentage endorsements within each category. *p* < 0.05

For frequency of use of student-generated resources, most students from both schools used online content generated by other students (17/31, 54.9% ATSU students; 18/26, 69.2% EVMS students) (Table 6). The ATSU students most frequently used self-generated study resources (20/30, 66.7%) and used this resource more often than EVMS students (10/27, 37.0%, *p* = 0.047) (Fig. 5).

**Table 6:** Survey responses of second-year medical students for frequency of use of student-generated resources

Frequency of Use of Student-Generated Resources	No. (%)						*p* value
	Never	Occasionally But Less Than Once a Month	About Once a Month	About Once a Week	Several Times a Week	Daily	
Study resources (e.g., flashcards, charts, notes) generated by other students							
ATSU (*n* = 31)	5 (16.1)	9 (29.0)	2 (6.5)	4 (12.9)	4 (12.9)	7 (22.6)	0.82
EVMS (*n* = 26)	7 (26.9)	4 (15.4)	3 (11.5)	2 (7.7)	3 (11.5)	7 (26.9)	
Practice questions generated by other students							
ATSU (*n* = 29)	15 (51.7)	7 (24.1)	0 (0)	4 (13.8)	2 (6.9)	1 (3.4)	0.87
EVMS (*n* = 27)	14 (51.9)	5 (18.5)	3 (11.1)	1 (3.7)	3 (11.1)	1 (3.7)	
Online content generated by other students (e.g., Anki cards)							
ATSU (*n* = 31)	6 (19.4)	3 (9.7)	2 (6.5)	3 (9.7)	3 (9.7)	14 (45.2)	0.49
EVMS (*n* = 26)	4 (15.4)	1 (3.8)	1 (3.8)	2 (7.7)	4 (15.4)	14 (53.8)	
Self-generated study resources (e.g., flashcards, charts, notes)							
ATSU (*n* = 30)	1 (3.3)	3 (10.0)	1 (3.3)	5 (16.7)	8 (26.7)	12 (40.0)	0.047
EVMS (*n* = 27)	7 (25.9)	4 (14.8)	2 (7.4)	4 (14.8)	2 (7.4)	8 (29.6)	
Self-generated online study resources (e.g., Anki cards)							
ATSU (*n* = 30)	8 (26.7)	4 (13.3)	1 (3.3)	2 (6.7)	2 (6.7)	13 (43.3)	0.18
EVMS (*n* = 27)	10 (37.0)	2 (7.4)	3 (11.1)	3 (11.1)	4 (14.8)	5 (18.5)	

*ATSU* A.T. Still University’s School of Osteopathic Medicine in Arizona, *EVMS* Eastern Virginia Medical School

### Use of Outside Resources Based on Discipline

For frequency of use by discipline, most students from both schools used outside resources to study pathology (17/31, 54.8% ATSU students; 26/27, 96.3% EVMS students), pathophysiology (18/31, 58.1% ATSU students; 27/27, 100% EVMS students), physiology (18/31, 58.1% ATSU students; 25/27, 92.6% EVMS students), and pharmacology (22/31, 71.0% ATSU students; 20/27, 74.1% EVMS students) (Table 7). Differences were found between the two groups for these four disciplines (all *p* < 0.046). For ATSU students, outside resources were most frequently used to study pharmacology and clinical sciences (21/31, 67.7%). For EVMS students, outside resources were most frequently used to study pathophysiology and pathology. Differences were also found between the two groups for the disciplines of biochemistry, genetics/molecular biology, and osteopathic principles and practice (all *p* < 0.02).

**Table 7:** Survey responses of second-year medical students for use of outside resources for specific disciplines

Frequency of use of outside resources for specific disciplines	No. (%)						*p* value
	Never	Occasionally but less than once a month	About once a month	About once a week	Several times a week	Daily	
Anatomy							
ATSU (*n* = 31)	2 (6.5)	4 (12.9)	3 (9.7)	11 (35.5)	8 (25.8)	3 (9.7)	0.07
EVMS (*n* = 27)	0 (0)	2 (7.4)	5 (18.5)	5 (18.5)	5 (18.5)	10 (37.0)	
Embryology							
ATSU (*n* = 31)	10 (32.3)	5 (16.1)	6 (19.4)	5 (16.1)	3 (9.7)	2 (6.5)	< 0.001
EVMS (*n* = 27)	1 (3.7)	2 (7.4)	3 (11.1)	4 (14.8)	5 (18.5)	12 (44.4)	
Biochemistry							
ATSU (*n* = 31)	3 (9.7)	5 (16.1)	6 (19.4)	5 (16.1)	9 (29.0)	3 (9.7)	0.02
EVMS (*n* = 27)	0 (0)	0 (0)	6 (22.2)	7 (25.9)	5 (18.5)	9 (33.3)	
Clinical sciences							
ATSU (*n* = 31)	2 (6.5)	1 (3.2)	3 (9.7)	4 (12.9)	13 (41.9)	8 (25.8)	0.52
EVMS (*n* = 27)	1 (3.7)	2 (7.4)	5 (18.5)	0 (0)	8 (29.6)	11 (40.7)	
Genetics/molecular biology							
ATSU (*n* = 31)	6 (19.4)	5 (16.1)	5 (16.1)	7 (22.6)	7 (22.6)	1 (3.2)	0.01
EVMS (*n* = 27)	0 (0)	0 (0)	4 (14.8)	4 (14.8)	8 (29.6)	11 (40.7)	
Osteopathic principles and practice (if applicable)							
ATSU (*n* = 31)	9 (29.0)	12 (38.7)	7 (22.6)	2 (6.5)	0 (0)	1 (3.2)	0.01
EVMS (*n* = 7)	0 (0)	0 (0)	0 (0)	0 (0)	2 (28.6)	5 (71.4)	
Microbiology/immunology							
ATSU (*n* = 31)	2 (6.5)	3 (9.7)	3 (9.7)	9 (29.0)	9 (29.0)	5 (16.1)	0.01
EVMS (*n* = 26)	0 (0)	0 (0)	2 (7.7)	1 (3.8)	9 (34.6)	14 (53.8)	
Pathology							
ATSU (*n* = 31)	1 (3.2)	1 (3.2)	2 (6.5)	10 (32.3)	11 (35.5)	6 (19.4)	0.01
EVMS (*n* = 27)	0 (0)	0 (0)	0 (0)	1 (3.7)	9 (33.3)	17 (63.0)	
Pathophysiology							
ATSU (*n* = 31)	2 (6.5)	1 (3.2)	2 (6.5)	8 (25.8)	12 (38.7)	6 (19.4)	0.01
EVMS (*n* = 27)	0 (0)	0 (0)	0 (0)	0 (0)	9 (33.3)	18 (66.7)	
Physiology							
ATSU (*n* = 31)	3 (9.7)	2 (6.5)	4 (12.9)	4 (12.9)	15 (48.4)	3 (9.7)	0.01
EVMS (*n* = 27)	0 (0)	0 (0)	0 (0)	2 (7.4)	10 (37.0)	15 (55.6)	
Pharmacology							
ATSU (*n* = 31)	4 (12.9)	1 (3.2)	0 (0)	4 (12.9)	16 (51.6)	6 (19.4)	0.046
EVMS (*n* = 27)	2 (7.4)	0 (0)	2 (7.4)	3 (11.1)	5 (18.5)	15 (55.6)	

*ATSU* A.T. Still University’s School of Osteopathic Medicine in Arizona, *EVMS* Eastern Virginia Medical School

### Qualitative Survey Responses

Fourteen students from ATSU and EVMS (21.5%) replied to the two open-ended survey questions. Five themes were identified: (1) distrust of current curriculum, (2) augmentation of instructor-created content, (3) preparation for clinical responsibilities, (4) creation of a framework for current and future learning, and (5) efficiency (Table 8). The themes of augmentation of instructor-created content, preparation for clinical responsibilities, and creation of a framework for current and future learning correspond to the quantitative analysis.

**Table 8:** Survey responses of second-year medical students (*N* = 14) to open-ended questions

Theme	Definition of theme	Representative quotes
Distrust of current curriculum	The feeling that the current curriculum does not address the needs of the student	Student uses outside resources “to verify accuracy of concepts taught in lecture.” “I wish our curriculum focused more on board-reared materials. I only reference class material because it most directly relates to the school-generated exams. But I do not believe our school’s questions relates [sic] to the board exams and wish there was a standard metric to better reflect board exams so as to be compared across universities.”
Augmentation of instructor-created content	The process of using outside resources to further clarify and amplify content and concepts to ensure learning	“I use outside resources if I feel like I don’t have a comprehensive understanding based off of what we’ve been taught in classes. If our coursework is comprehensive, I wouldn’t find the need to use outside resources.” “Outside resources are great for supplemental learning in addition to instructor’s lectures. I tend to learn better when I’m able to see a concept explained from different angles, so having both lectures and outside resources to learn from greatly improves my studying.”
Preparation for clinical responsibilities	The use of outside resources to ensure appropriate coverage of clinically relevant content that is important for the transition to clerkships	Student uses outside resources for “preparing for rotations, answering pimping questions from preceptors.” “Academic faculty teaching the majority of lectures has [sic] an extremely poor understanding of the scope to which medical students need to study their material. Ultimately the focus of medical education should be towards the future clinical practice. The majority of the faculty who are PhDs in their respective fields are extremely knowledgeable but their knowledge is purely theoretical. Lectures delivered vary wildly in quality and tend to focus on minutia which is interesting to the professor and has extremely low yield value for medical professional exams.”
Creation of a framework for current and future learning	The use of outside resources to ensure cognitive scaffolding that allows the student to connect content and concepts to the big picture	“I wouldn’t fully understand concepts with most subject [sic] taught at [blinded] without the help of effective outside resources.” “Some professors go really far into the weeds on little aspects of things and don’t clarify the big picture idea, so outside resources are utilized to help with that.”
Efficiency	The use of outside resources to complete learning tasks successfully without wasting time or energy	“More efficient and smarter, time wise” (in reference to use of outside resources) “Concise, effective teaching of high yield topics” (in reference to why they use outside resources)

## Discussion

In the current study, we evaluated medical student resource use at two institutions. Our results suggested statistically significant differences between the two medical schools included in our analyses and were corroborated by existing studies on resource use [5, 19, 20]. More specifically, the choices of students who responded to our survey indicated resource use was shaped by academic environment. Students reported that their use of outside resources was often influenced by academic support staff and interactions with students from other medical schools and that they most often used live lecture recordings, transcripts, instructor-led review sessions, and self-generated study resources. At EVMS, there are required and optional sessions students attend that discuss methods for preparing for licensing and course exams. Because these findings suggest a student’s institution influences their resource use decisions, there should be alignment between student advising and the medical education program. An emerging interest in learning communities provides one example of a curricular structure that emphasizes advising while scaffolding curricular experiences that could be an effective way to support effective student use of external resources [21]. While our institutions have not formally adopted this model, it would provide an interesting subject for future investigation.

Results from the current study also suggested the observed differences in student behaviors between the two groups were related to the types of assessment and influence of organizational features (such as student affairs) in the curriculum. Differences in the learning environments, such as using instructor-created tests versus retired board review exam questions, providing access to student academic support offices, and recording and reporting student rank despite an existing pass-fail grading system, likely explain these results and support existing trends. Overall, these results emphasize the importance of local contextual features and suggest that medical school administrators should consider their organizational culture beyond day-to-day student and faculty interactions [22]. As medical schools continue to assess the evolving integration of student affairs activities and staff as curricular partners, there is the opportunity to promote personalized, student-centered approaches that can aid in the development of SRL and effective learning behaviors [23]. Additionally, the incorporation of NBME-generated exams as part of the medical school curriculum will motivate learners to use outside resources to improve exam performance, and create a framework for current and future learning [4].

Although EVMS students were more likely to use outside resources on a regular basis, quantitative and qualitative results suggest both groups of students frequently used outside resources to study, which highlighted the importance of supplementary materials for augmenting student learning. As learning experiences and the learning environment are influenced by multiple factors [24], students have become motivated to access the resources they perceive will help them achieve passing scores on licensing exams or support their long-term goals (such as specialty choice). Additionally, the prevalence of digital tools and influence of peer advice are highly influential in students’ decision to use outside resources [25, 26]. Therefore, the medical education community must recognize and co-create the best environment with the best resources for maximum impact on our students [24].

Increasingly, students believe the medical education curriculum inaccurately represents the concepts tested on board exams and the knowledge required to practice medicine. For example, Khalil et al. found that student perceptions of curriculum deficits are associated with lower performance on the USMLE Step 1 exam [27]. Additionally, students often prioritize exam preparation over engaging with the prescribed preclinical curriculum, thus raising questions about the value of instructor-created content [28]. Results of our qualitative analysis supported this belief; distrust of the current curriculum was one of our five identified themes. Only through active engagement can we address this growing level of student distrust with the medical education curriculum. A good place to start is at the local level. Research has shown that students’ coursework grades are most predictive of success on the United States Medical Licensing Examination Step 1 and Step 2 exams [29]. Therefore, medical schools can demonstrate the alignment of their curriculum by stressing the relationships between instructor-led content and resources, internal tests, and licensing examination outcomes. Although future research is needed to explore the root cause of student distrust, discussions around incorporating flexibility and reducing overscheduling have been proposed to regain trust [30].

Another interesting result from our study was the more prominent use of self-generated study resources by ATSU students than by EVMS students. Given the current focus on self-directed learning for Liaison Committee on Medical Education accreditation [31] and the importance of self-regulated learning (SRL) as a measurable outcome, educators should provide opportunities for intentional practice that incorporate the four elements of SRL (planning, learning, assessment, and adjustment) [32]. Based on our study results, students who generated their own study resources seemed to be following the planning and learning elements of SRL by deciding what they needed to learn and how they needed to learn it. Although we did not explicitly measure how students modified their use of self-generated resources, their frequent use of these resources suggested they were also assessing whether they had learned what they intended to and were adjusting as necessary. Since students seem likely to use self-generated resources during their education, medical schools should support this practice by encouraging discussions about the SRL process and providing a framework to help students cultivate this skill set as they enter semiautonomous practice in residency [32].

In the current study, comparisons of the frequency of use of outside resources by discipline highlighted major differences between the two groups of students, particularly for basic science disciplines with the exception of anatomy. The similar findings between groups for anatomy may be related to how this discipline is taught. For example, both schools predominantly incorporate anatomy into the first-year curriculum and, since there tends to be less anatomy content in the second-year curriculum, the reported use of resources was similar. The differences in the frequency of use of external resources for other basic science disciplines are likely multi-factorial. Medical students increasingly rely on external resources to supplement their learning, driven by the need for efficiency, clarity, and exam preparation [12, 33, 34]. While traditional resources like lectures and textbooks remain important for learning new material, students frequently use online question banks and videos for revision [35]. Given this finding, medical schools should consider providing additional resources to augment medical student learning. Ready availability of such content could help students build foundational knowledge of the topic while enhancing opportunities for student choice and use of resources that enhance their learning.

The current study has a few limitations. The responses rates of 19% at EVMS and 20% at ATSU may limit the generalizability of results. Further, the self-reported nature of our survey may have introduced inaccuracies or bias. Although our study investigated resource use among students at two institutions, the correlation between resource use and academic outcomes was not directly investigated. However, other studies have reported that resource use and academic outcomes are related [4, 15, 29]. Our results supported this relationship and highlighted the importance of evaluating why students decide to use outside resources, what resources they use, and how they actually use them. Ultimately, when we understand student decisions about resource use, we can promote activities and resources that support SRL and better academic outcomes.

## Conclusions

As suggested by results of the current study, variations in resource use patterns exist among students from different medical schools and may be explained by institutional differences in the academic environment, organizational culture, and developmental path of medical students. Given these results, medical schools should consider developing strategies that better support their students through tailored guidance, curricular adjustments, and resource navigation of all available learning options. By recognizing and addressing these factors, medical schools can optimize their educational strategies and enhance overall student learning experiences across academic disciplines for best alignment with their curricular goals and intended student outcomes.

## Supplementary Information

Below is the link to the electronic supplementary material.

Supplementary file1 (PDF 807 KB)

## Data Availability

The datasets used and/or analyzed during the current study are available from the corresponding author upon reasonable request.

## Abbreviations

ATSU: A.T. Still University’s School of Osteopathic Medicine in Arizona
EVMS: Eastern Virginia Medical School
NBME: National Board of Medical Examiners
SRL: Self-regulated learning
