# Supplementary material for: An Investigation of Second-Year Medical Students’ Use of Outside Resources at Two Institutions
Source: Med Sci Educ. 2024 Dec 6;35(2):847–62. doi: 10.1007/s40670-024-02243-1 (PMC12058637; doi:10.1007/s40670-024-02243-1)
Supplement: Supplementary file 1 — Supplementary file1 (PDF 807 KB) [file 40670_2024_2243_MOESM1_ESM.pdf]

Default Question Block

This survey is for research conducted by the School of Osteopathic Medicine in Arizona (SOMA) at A. T. Still University, Eastern Virginia Medical School (EVMS) and University of Central Florida College of Medicine (UCF COM). The purpose of this study is to increase understanding of student resource use across multiple institutions (both osteopathic and allopathic). Your participation is voluntary and will only involve completing the questionnaire. Completing this questionnaire should require 15 minutes of your time. Please do not use any outside assistance while answering these questions. Please answer each question honestly and to the best of your ability. No names or other identifying information will be used in discussing or reporting data. The results of this research might be published. Any research reports or publications resulting from this research will not reveal your name or identity. All of your data will remain confidential. There are no direct benefits to you for completing this questionnaire. By completing the questionnaire, you agree to allow your survey responses to be used for this study. Your participation is voluntary, and you may refuse to complete the questionnaire or stop at any time if you wish.

How likely are you to use outside resources (not required for class) recommended by each of the following sources?

	Not at all	Slightly	Somewhat	Very	Extremely
Instructors	○	○	○	○	○
Classmates or other medical students	○	○	○	○	○
Students at other medical schools	○	○	○	○	○
Residents	○	○	○	○	○
Academic support staff	○	○	○	○	○
Internet Resources (Google or Internet Forum)	○	○	○	○	○
Library	○	○	○	○	○
Other [ ]	○	○	○	○	○

What is the likelihood of using outside resources to:

	Almost always true	Usually true	Occasionally true	Usually not true	Almost never true
prepare for licensing exams e.g. COMLEX 1, USMLE Step 1 or 2	○	○	○	○	○
prepare for course exams	○	○	○	○	○
seek efficient means of comprehending concepts	○	○	○	○	○
seek effective learning resources	○	○	○	○	○
provide more detail than course material	○	○	○	○	○
access practice questions	○	○	○	○	○
have a framework for understanding content	○	○	○	○	○
have an interactive experience	○	○	○	○	○
receive feedback	○	○	○	○	○
have opportunities for practice	○	○	○	○	○

Are there other reasons not mentioned above that you use outside resources? Please explain.

Please indicate which of the following categories most closely approximates the frequency of your use of each of the following resources.

	Never	Occasionally, but less than once a month	About once a month	About once a week	Several times a week	Daily	NA
Instructor slides	○	○	○	○	○	○	○
Live lecture recordings	○	○	○	○	○	○	○

	Never	Occasionally, but less than once a month	About once a month	About once a week	Several times a week	Daily	NA
Transcripts	○	○	○	○	○	○	○
Pre-recorded presentations	○	○	○	○	○	○	○
Practice questions provided by the instructor	○	○	○	○	○	○	○
Assigned or recommended reading (textbook or articles)	○	○	○	○	○	○	○
Other instructor created resources (study guides, lab handouts, etc.)	○	○	○	○	○	○	○
Office hours or other instructor interactions	○	○	○	○	○	○	○
Instructor Led Review sessions	○	○	○	○	○	○	○
Supplemental textbooks or articles (not assigned by the instructor)	○	○	○	○	○	○	○
General search engines (ex. Google)	○	○	○	○	○	○	○
Literature Database (ex PubMed)	○	○	○	○	○	○	○
Library Website search	○	○	○	○	○	○	○
Online videos (ex. YouTube)	○	○	○	○	○	○	○
Streaming Media (Procedures Consult, Acland’s Video Atlas, Bates)	○	○	○	○	○	○	○
Wikipedia	○	○	○	○	○	○	○
Apps (ex UpToDate or Dynamed)	○	○	○	○	○	○	○
Board review resources (Kaplan, First Aid, Sketchy micro/pharm, Pathoma)	○	○	○	○	○	○	○
Practice Questions provided Board review question banks (Kaplan,Uworld,COMBank, BoardVitals)	○	○	○	○	○	○	○

	Never	Occasionally, but less than once a month	About once a month	About once a week	Several times a week	Daily	NA
3D models (printed 3D model or virtual 3D model)	○	○	○	○	○	○	○
Study resources (ex. flashcards, charts, or notes) generated by other students	○	○	○	○	○	○	○
Practice questions generated by other students	○	○	○	○	○	○	○
Online content generated by other students (ex. Anki cards)	○	○	○	○	○	○	○
Self-generated study resources (flashcards, charts, notes, etc.)	○	○	○	○	○	○	○
Self-generated online study resources (ex. Anki cards)	○	○	○	○	○	○	○

How often do you use outside resources for the following disciplines?

	Never	Occasionally, but less than once a month	About once a month	About once a week	Several times a week	Daily	NA
Anatomy	○	○	○	○	○	○	○
Embryology	○	○	○	○	○	○	○
Biochemistry	○	○	○	○	○	○	○
Clinical Sciences	○	○	○	○	○	○	○
Genetics/Molecular Biology	○	○	○	○	○	○	○
Osteopathic Principles and Practice (if applicable)	○	○	○	○	○	○	○
Microbiology/Immunology	○	○	○	○	○	○	○
Pathology	○	○	○	○	○	○	○
Pathophysiology	○	○	○	○	○	○	○
Physiology	○	○	○	○	○	○	○

	Never	Occasionally, but less than once a month	About once a month	About once a week	Several times a week	Daily	NA
Pharmacology	○	○	○	○	○	○	○

Please provide any additional comments with regards to use of outside resources.

Select the institution where you are a student:

- ○ A.T. Still University
- ○ Eastern Virginia Medical School
- ○ University of Central Florida College of Medicine

Your age:

- ☐ 20 - 25 years old
- ☐ 26- 30 years old
- ☐ Over 30 years old

Your sex:

- ○ Man
- ○ Woman
- ○ Non-Binary
- ○ Transgender
- ○ Prefer not to respond

What is your highest degree attained prior to beginning medical school?

- BA
- BS
- MS
- PhD
- Other

If you selected Other, what is your highest degree?

Powered by Qualtrics
